# Data on optimization of the non-linear Muskingum flood routing in Kardeh River using Goa algorithm

**DOI:** 10.1016/j.dib.2020.105398

**Published:** 2020-03-10

**Authors:** Saeid Khalifeh, Kazem Esmaili, SaeedReza Khodashenas, Saeid Akbarifard

**Affiliations:** a Water Science and Engineering, Faculty of Agriculture, Ferdowsi University of Mashhad, Mashhad, Iran; b Department of Water Science and Engineering, Faculty of Agriculture, Ferdowsi University of Mashhad, Mashhad, Iran; c Water Resources Engineering, Department of Hydrology and Water Resources, Faculty of Water Sciences Engineering, Shahid Chamran University of Ahvaz, Ahvaz, Iran

**Keywords:** Muskingum, Kardeh, Non-linear, Grasshopper optimization algorithm

## Abstract

This article describes the time series data for optimizing the Non-linear Muskingum flood routing of the Kardeh River, located in Northeastern of Iran for a period of 2 days (from 27 April 1992 to 28 April 1992). The utilized time-series data included river inflow, Storage volume and river outflow. In this data article, a model based on the Grasshopper Optimization Algorithm (GOA) was developed for the optimization of the Non-linear Muskingum flood routing model. The GOA algorithm was compared with other metaheuristic algorithms such as the Genetic Algorithm (GA) and Harmony search (HS). The analysis showed that the best solutions achieved by the GOA, Genetic Algorithm (GA), and Harmony search (HS) were 3.53, 5.29, and 5.69, respectively. The analysis of these datasets revealed that the GOA algorithm was superior to GA and HS algorithms for the optimal flood routing river problem.

**Specifications table**

**Table:** 

Subject	Water Resources Management
Specific subject area	River Engineering; Hydrology and Water Resources; Metaheuristic Algorithms
Type of data	Table and figures
How the data were acquired	Hydrological Measurement obtained raw data, and the data analyzed were derived from the MATLAB software.
Data format	Raw and analyzed
Parameters for data collection	The daily time series of inflow in the river, and the daily time series of outflow in the river.
Description of data collection	The Khorasan Razavi Water Authority provides hydrological datasets
Data source location	The Kardeh River located in the Kardeh basin (36° 48´ E longitude, 59° 30´ N latitude), North Eastern of Iran.
Data accessibility	All processed data and raw data are available in this data article as a supplementary file.

## Value of the data

• Data on the volumes of river inflow and the volumes of river outflow in the Kardeh River provide an overview of the status of the river in the year of 1992.
• These data are used to determine floodplain and flood forecasting.
• These data can be used to analyze the water resources status in the Kardeh River.
• The data will be useful for modeling purposes, especially relating to the Kardeh River status.
• The analysis obtained herein by Metaheuristic Algorithms (MAs) solver can serve as a standard benchmark for other researchers to compare their analysis of the other methods using this dataset.
• Other researchers can use the GOA algorithm in solving problems such as the flood routing in the river with confidently.

## 1 Data

Flood routing is one of the most complex problems that is investigated in open channel hydraulics and river engineering. It can help design engineers to recognize the impacts of riverine projects. Among the different flood routing methods, the Muskingum model, as one of the most widely used the hydrologic methods of flood routing, has been widely used with high accuracy in river flood projects. Different researchers have considered the Parameters estimation of the Non-linear Muskingum food-routing model, and several methods have been utilized to this purpose [1].

**Fig. 1:**
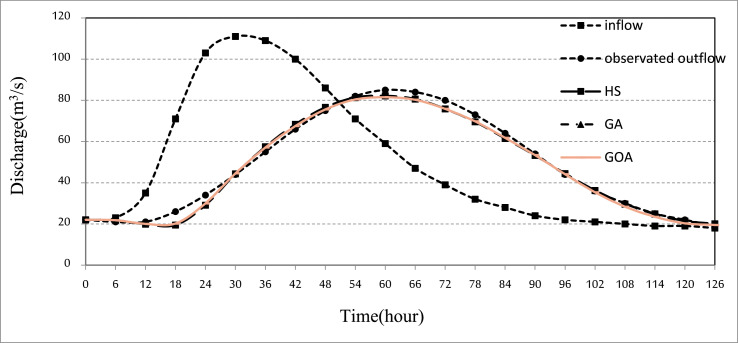
Time series chart of the dataset. The figure shows the time series hydrological dataset consists of river inflow, river observed outflow, and river outflow routed by the algorithms for the Wilson River.

In the present paper, Kardeh River flood routing is considered in terms of careful river management. The time series hydrological dataset consists of river inflow, Storage volume, and river outflow for a period of 2 days (from 27 April 1992 to 28 April 1992). The utilized data are shown in Fig. 1. River inflow is the volume of water inflow to the Wilson River, which is measured in cubic meters per second (m^3^/s).

**Fig. 2:**
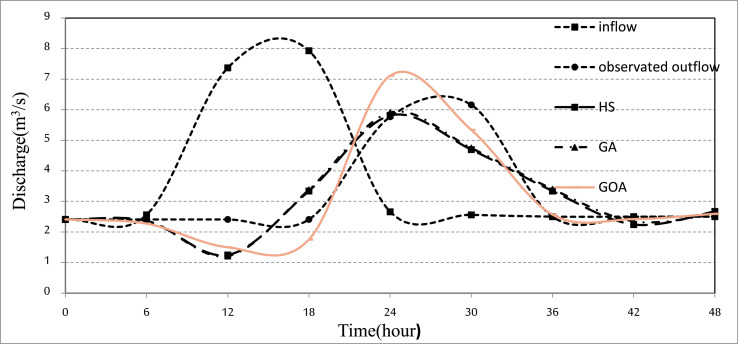
Time series chart of the dataset. The figure shows the time series hydrological dataset consists of river inflow, river observed outflow, and river outflow routed by algorithms for the Kardeh River for a period of 2days (from 27 April 1992 to 28 April 1992).

**Fig. 3:**
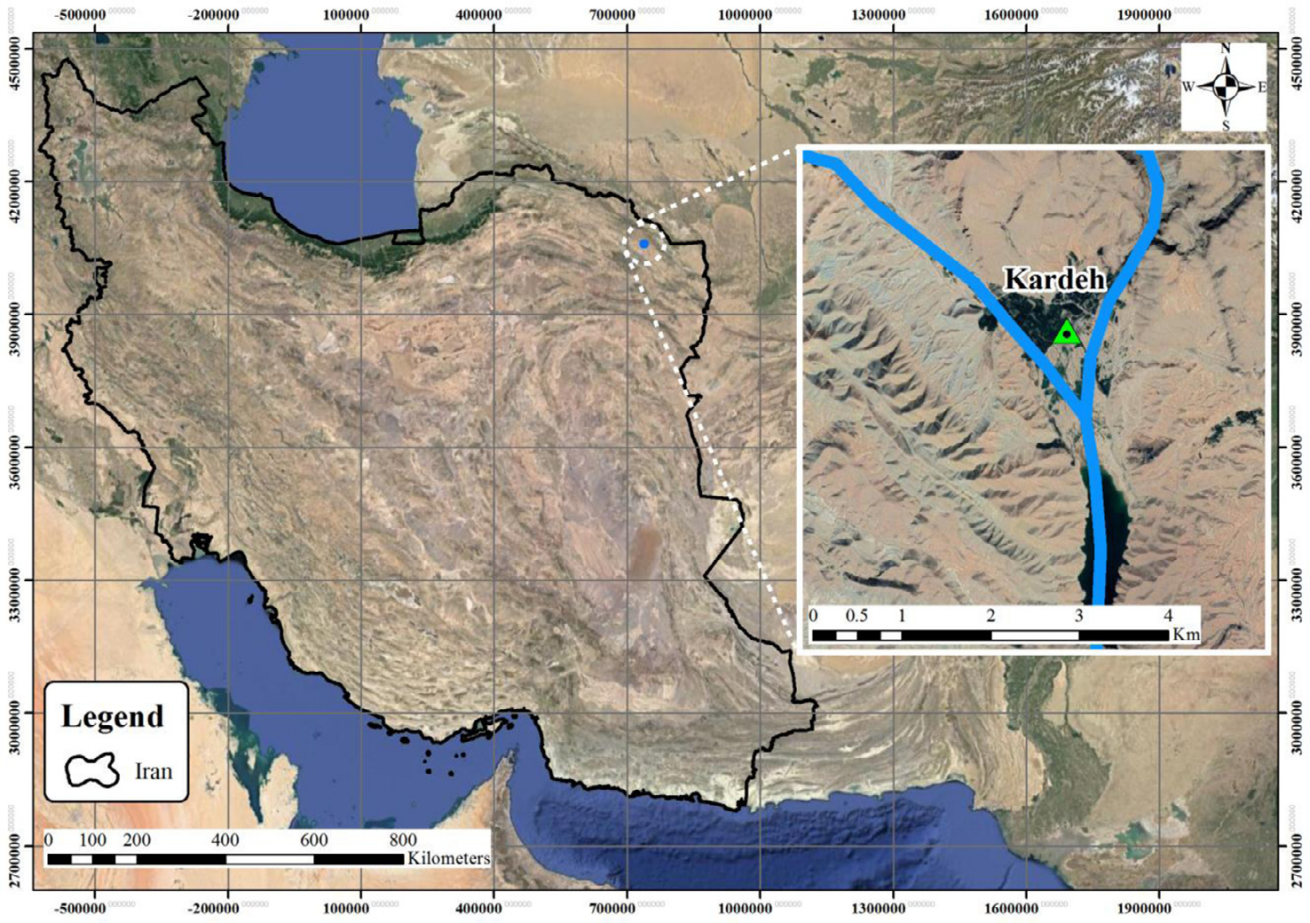
Location of the Kardeh River in the Kashafrood basin (Northeastern of Iran).

**Fig. 4:**
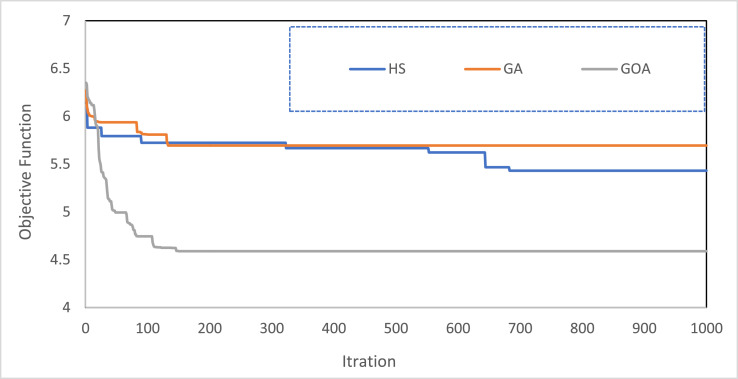
The convergence of applied algorithms in the Kardeh River. The figure shows the convergence rate of applied algorithms in reaching the optimum value for the flood routing problem. It also indicates the rapid convergence of the GOA in comparison with the other algorithms.

The Fig. 2 shows the time series hydrological dataset consists of river inflow, river observed outflow, and river outflow routed by the algorithms for the Kardeh River. Fig. 3 shows the location of the Kardeh River in the Kashafrood basin. Table 1 displays the values of used algorithms parameters for the flood routing problem. Table 2 describes the objective value of objective functions and the average CPU run time obtained by each algorithm for the Wilson River problem. Table 3 describes the objective value of objective functions and the average CPU run time obtained by each algorithm for the Kardeh River problem. Fig. 4 represents the convergence rate of the applied GOA algorithm in reaching the optimum value for 1000 iterations.

**Table 1:** Values of used algorithms parameters for problem.

**GOA**	**parameter**	**iterations**	**Number of variables**	**Number of search agents**	**–**	**–**
	**Value**	1000	3	100	–	–
**GA**	**parameter**	**iterations**	**Number of variables**	**Number of genes**	**Mutation rate**	**Crossover rate**
	**Value**	1000	3	100	0.01	0.8
**HS**	**parameter**	**iterations**	**Number of variables**	**Population Size**	**HMCR**	**PAR**
	**Value**	1000	3	100	0.1	0.1

**Table 2:** Analyses of 10 runs of the Wilson River. The objective value of objective functions and the average CPU run time for each algorithm were presented in this table for the Wilson River problem. Analysis of datasets in the table showed that GOA was able to produce superior solutions for the Wilson River.

Number of runs	GOA		HS		GA	
	Optimal value	CPU time (s)	Optimal value	CPU time (s)	Optimal value	CPU time (s)
**1**	182.65	18.69	131.24	3	130.33	8.52
**2**	192.82	19	129.44	2.89	134.88	7.98
**3**	128.7869	19.33	131.25	2.82	135.11	7.58
**4**	128.7887	19.5	130	2.80	131.87	7.23
**5**	128.7864	19.31	131.26	2.82	133.05	7.39
**6**	128.7865	19.61	133.11	2.84	129.67	7.64
**7**	128.7893	19.56	133.08	2.80	129.01	11.32
**8**	200.17	20.47	133.89	2.83	145.35	7.27
**9**	128.7866	19.52	137.51	2.81	128.9	7.13
**10**	128.7864	19.38	130.19	2.82	141.99	7.26
**Best**	**128.7864**		129.44		128.9	
**Worst**	**200.17**		137.51		1.9616	
**Average**	**147.7**		132.07		134.01	
**SD**	**30.75**		2.39		5.61	
**Coefficient of variation**	**0.2082**		0.018		0.041	
**Best CPU time (s)**	**18.69**		2.80		7.13

**Table 3:** Analyses of 10 runs of the Kardeh River. The objective value of objective functions and the average CPU run time for each algorithm were presented in this table for the Kardeh River problem. Analysis of datasets in the table showed that GOA was able to produce superior solutions for the Kardeh River.

Number of runs	GOA		HS		GA	
	Optimal value	CPU time (s)	Optimal value	CPU time (s)	Optimal value	CPU time (s)
**1**	3.6257	35.74	5.6	2.12	5.6940	8. 2
**2**	3.8024	35.88	5.57	1.97	5.6938	6.15
**3**	3.7048	36.28	5.69	1.94	5.6937	6.78
**4**	3.6021	35.61	5.7	2	5.6938	6.88
**5**	3.5348	35.99	5.29	1.99	5.6937	6.32
**6**	3.9208	35.73	5.47	1.83	5.6939	5.91
**7**	3.8350	35.79	5.48	1.85	5.6937	5.8
**8**	3.7434	35.76	5.7	1.96	5.6937	6.16
**9**	3.9821	35.71	5.7	1.85	5.6941	5.9
**10**	3.5644	36.17	5.44	1.84	5.6938	8.33
**Best**	**3.5348**		5.29		5.6937	
**Worst**	**3.9821**		5.7		5.6941	
**Average**	**3.7315**		5.5641		5.6938	
**SD**	**0.15**		0.14		0.00014	
**Coefficient of variation**	**0.0409**		0.025		0.000024	
**Best CPU time (s)**	**35.61**		2.80		5.8

## 2 Experimental design, materials, and methods

In this data article, using the time-series dataset, a model based on Grasshopper Optimization Algorithm (GOA) was developed for the optimization of the Non-linear Muskingum flood routing model. GOA Algorithm was first developed in the optimization of the flood routing model. The details of the GOA algorithm were provided by Saremi et al. (2017) [2]. The GOA algorithm was compared with other well-known developed evolutionary algorithms, including GA and HS algorithms [3], [4], [5], [6], [7]. It is noteworthy that all the metaheuristic algorithms were coded in MATLAB software.

### 2.1 Experimental design

The simulation-optimization model for producing a time-series dataset of the outflow of the Kardeh River was structured for a 2-days flood with a 6 h’ time step.

In this paper, the Objective function was used in the form of minimizing the sum of squares of residuals (SSQ) between actual and routed outputs according to Eq. (1) to estimate the optimal values of K, X and m parameters in the Muskingum model.

Objective functions and constraints of the Kardeh River are as follows:

(1) $$Min\left(SSQ\right)=\sum_{t=1}^{N}{\left(O_{t}-O_{ct}\right)}^{2}$$

(2) $$\frac{ds}{dt}=I_{t}-O_{t}$$

(2) $$S_{t}=K\left[XI_{t}+\left(1-X\right)O_{t}\right]$$

(3) $$S_{t}=K{\left[XI_{t}+\left(1-X\right)O_{t}\right]}^{m}$$

(4) $$S_{t}=K\left[X{I_{t}}^{m}+\left(1-X\right){O_{t}}^{m}\right]$$

(5) $$O_{t}=\left(\frac{1}{1-X}\right){\left(\frac{S_{t}}{K}\right)}^{\frac{1}{m}}-\left(\frac{X}{1-X}\right)I_{t}$$

(6) $$\frac{\Delta s_{t}}{\Delta t}=-\left(\frac{1}{1-X}\right){\left(\frac{S_{t}}{K}\right)}^{\frac{1}{m}}+\left(\frac{1}{1-X}\right)I_{t}$$

(7) $$S_{t+1}=S_{t}+\Delta S_{t}$$

Where; S_t_ [L^3^] = simultaneous amounts of storage, I_t_ [L^3^/T] = inflow, O_t_ [L^3^/T] = outflow, O_ct_ [L^3^/T] = outflow routed, at time = t, K [L^3 (1-m)^.T^m^] =storage-time constant and is greater than 0, X = weighting factor usually varying between 0 and 0.5 [8].

The model is easy to use, requiring the assessment of two parameters (K, X, m), which can be simply obtained by observed inflow and outflow data. Flood routing is a component of the rainfall-run off transformation process. In rainfall-runoff modeling, Non-linear responses are primarily attributable to two causes. The most important is the effect of antecedent conditions: the wetter the catchment before a unit input of rainfall, the greater the volume of runoff that will be generated. Thus, the relationships between total rainfall and runoff are generally considered to be Non-linear [9], [10], [11].

The secondary cause of Non-linearity is attributable to the change of flow velocity with discharge.

In general, average flow velocities increase with the flow in a Non-linear way, and the relationship between the weighted flow and the storage is Non-linear. Thus, using the linear form of the Muskingum model may introduce considerable error (Yoon and Padmanabhan) [9]. For this purpose, Gill [10] suggested two Non-linear Muskingum models given as subsequently Eqs. (3) and (4).

Where m = an exponent for considering the effects of Non-linearity and is greater than 1 for Non-linear models (the original linear model can be a special case of the Non-linear model where *m* = 1). In the Model, K, X and m are unknown parameters, and S_t_ and O_t_ must be handled as Non-negative variables.

This model has an additional parameter compared to Eq. (2). The standard procedure for applying the Muskingum method involves two basic steps: calibration and prediction. In the calibration step, the parameter values for the Muskingum model of a river reach are determined by using historical inflow-outflow hydrograph. The prediction step is the solution of a routing problem in which the outflow hydrograph for a given inflow hydrograph is determined by using the routing equations. The derivation of the routing equation for the Non-linear Muskingum model is straight forward. By rearranging the Non-linear Muskingum equation, the rate of outflow Q_t_, at time t, can be expressed in terms of channel storage, St and inflow rate, It, as Eqs. (5) and (6).

### 2.2 Analysis of datasets

The analyses of this data article showed that the best solution Parameters achieved by the GOA, GA and HS algorithm for the Kardeh River as a dataset problem were 3.53, 5.29 and 5.69, respectively. The analyses revealed that the GOA algorithm was the superior algorithm in the optimal operation of the Kardeh River.

All analyses of this research for each algorithm are presented in Tables 2 and 3 and Fig. 1, Fig. 2, Fig. 3.

## Data availability statement

All models, datasets, or codes generated or used during the article are available from the corresponding author by request.

## Conflict of Interest

The authors declare that they have no known competing financial interests or personal relationships that could have appeared to influence the work reported in this paper.

## Appendix Supplementary materials

Image, application 1
